# A low-cost and versatile paramagnetic bead DNA extraction method for *Mycobacterium ulcerans* environmental surveillance

**DOI:** 10.1128/aem.01021-24

**Published:** 2024-09-10

**Authors:** Jean Y. H. Lee, Jessica L. Porter, Emma C. Hobbs, Pam Whiteley, Andrew H. Buultjens, Timothy P. Stinear

**Affiliations:** 1 Department of Microbiology and Immunology, The University of Melbourne at The Doherty Institute for Infection and Immunity, Victoria, Australia; 2 Department of Infectious Diseases, Monash Health, Clayton, Victoria, Australia; 3 Melbourne Veterinary School, Faculty of Science, The University of Melbourne, Werribee, Victoria, Australia; 4 World Health Organisation Collaborating Centre for Mycobacterium ulcerans, Victorian Infectious Diseases Laboratory, Doherty Institute, Melbourne Health, Melbourne, Victoria, Australia; Centers for Disease Control and Prevention, Atlanta, Georgia, USA

**Keywords:** *Mycobacterium ulcerans*, Buruli ulcer, DNA extraction, paramagnetic beads, Australian native possums

## Abstract

In Australia, native possums are a major wildlife reservoir for *Mycobacterium ulcerans*, the causative agent of the neglected tropical skin disease Buruli ulcer (BU). Large-scale possum excreta surveys that use PCR to detect *M. ulcerans* in 100–1,000 s of excreta specimens are an important tool that can inform geospatial modeling and predict locations of future human BU risk. However, the significant expense of commercial kits used to extract DNA from specimens is a major barrier to routine implementation. Here, we developed a low-cost method for DNA extraction from possum excreta, possum tissue, and pure mycobacterial cultures, using a guanidinium isothiocyanate lysis solution and paramagnetic beads. In a 96-well plate format for high-throughput processing, the paramagnetic bead DNA extraction method was threefold less sensitive but only 1/6 the cost of a commonly used commercial kit. Applied to tissue swabs, the method was fourfold more sensitive and 1/5 the cost of a commercial kit. When used for preparing DNA from pure mycobacterial cultures, the method yielded purified genomic DNA with quality metrics comparable to more lengthy techniques. Our paramagnetic bead method is an economical means to undertake large-scale *M. ulcerans* environmental surveillance that will directly inform efforts to halt the spread of BU in Victoria, Australia, with potential for applicability in other endemic countries.

**IMPORTANCE:**

Buruli ulcer (BU) is a neglected tropical skin disease, with an incidence that has dramatically increased in temperate southeastern Australia over the last decade. In southeastern Australia, BU is a zoonosis with native possums the major wildlife reservoir of the causative pathogen, *Mycobacterium ulcerans*. Infected possums shed *M. ulcerans* in their excreta, and excreta surveys using PCR to screen for the presence of pathogen DNA are a powerful means to predict future areas of Buruli ulcer risk for humans. However, excreta surveys across large geographic areas require testing of many thousands of samples. The cost of commercial DNA extraction reagents used for preparing samples for PCR testing can thus become prohibitive to effective surveillance. Here, we describe a simple, low-cost method for extracting DNA from possum excreta using paramagnetic beads. The method is versatile and adaptable to a variety of other sample types including swabs collected from possum tissues and pure cultures of mycobacteria.

## INTRODUCTION

Buruli ulcer (BU) is a bacterial infection affecting subcutaneous tissues, caused by *Mycobacterium ulcerans*. Reported in 33 countries across Africa, the Americas, Asia, and the Western Pacific, BU is classified by the World Health Organization as a neglected tropical skin disease (1–3). Although Sub-Saharan Africa has the highest global burden of disease, of the countries that report annual suspected case numbers, Australia was second only to the Democratic Republic of the Congo in 2022, with 343 and 558 new reported cases, respectively (4, 5). In temperate southeastern Australia, over the last 26 years, the endemic region for BU has expanded from selected coastal regions in the state of Victoria to the major metropolitan centers of Geelong and Melbourne, with state-wide case numbers escalating to 362 notifications in 2023, the highest since the disease became notifiable to the Victorian Department of Health in 2004 (6, 7). Extensive environmental field studies in this region have led to the discovery that BU is a zoonotic infection, with Australian native possums, a wildlife reservoir of *M. ulcerans*, and mosquitoes, a primary vector for transmission to humans (8–13). In Africa, a *M. ulcerans* wildlife reservoir analogous to possums is yet to be discovered, and while there is a lack of direct evidence implicating mosquitos as a vector, local studies suggest a role for aquatic insects spreading the bacterium (14–17).

While *M. ulcerans* infection has been reported in both native Australian (11, 13, 18–20) and domesticated animal species both in Australia and overseas (1, 19, 21–23), two possum species, the common ringtail (*Pseudocheirus peregrinus*) and common brushtail (*Trichosurus vulpecula*) possums, have been identified as major reservoirs for *M. ulcerans* infection in Victoria, Australia (9–13). The percentage of affected possums varies by region. In the endemic area of Point Lonsdale, a 2010 study reported 38% (*N* = 16/42) of ringtail possums and 24% (*N* = 5/21) of brushtail possums to have either laboratory-confirmed *M. ulcerans* lesions or *M. ulcerans* PCR-positive excreta (11). This study observed that *M. ulcerans* DNA was detected in 41% of possum fecal samples collected in a Buruli endemic area compared with less than 1% of fecal samples collected from non-endemic areas (*P* < 0.0001) (11). A 2014 study performed in the Mornington Peninsula detected *M. ulcerans* in 9.3% (*N* = 20/216) of ringtail and 66.6% (*N* = 4/6) of brushtail possum excreta sampled (9). This study also demonstrated a statistically significant (*P* < 0.0001), non-random clustering of *M. ulcerans*-positive possum excreta within a 0.42-km radius of the addresses of confirmed human cases of BU (9). The hypothesis that possums are a sentinel species that can be used to predict human BU cases prompted more sophisticated confirmatory studies (8, 10). Most recently, a 2024 study by Mee et al. used spatial scanning statistics to demonstrate overlap between *Aedes notoscriptus* mosquitos that tested positive for *M. ulcerans*, with clusters of native possums shedding *M. ulcerans*, and human cases of BU (10). Using targeted bacterial genome enrichment, a transmission chain between mosquitos, possums, and humans was established. Additionally, metabarcoding mosquito blood-meal analyses demonstrated the feeding of individual mosquitos on both possums and humans providing a potential transmission pathway from infected possums to humans (10).

Despite recent advances in understanding the transmission of *M. ulcerans*, studies of the environmental ecology of this pathogen remain challenging, primarily due to its slow growth and concomitant difficulties isolating this organism in pure culture from microbially complex environmental samples (11, 24). A TaqMan quantitative polymerase chain reaction (qPCR) targeting IS*2404* has been the main methodology employed to infer the presence of *M. ulcerans* in the environment (8, 10–13). The IS*2404* insertion sequence encodes a 327-amino acid transposase (25) that recurs approximately 200 times in the *M. ulcerans* genome (25–27), making it an ideal target for PCR amplification. Prior to testing, possum excreta samples require DNA extraction; the cost of commercial DNA extraction kits can become prohibitively expensive in large-scale surveillance studies involving thousands of samples. To overcome this barrier, we sought to develop a significantly lower-cost DNA extraction method.

Australian brushtail and ringtail possums are predominantly folivores, with leaves of *Eucalyptus* species being their primary food source, supplemented by shrub foliage, flowers, and fruit, and in the case of brushtail possums, small quantities of animal material (28, 29). Subsequently, possum excreta are rich in plant matter (29), including humic substances, which are compounds resulting from the decomposition of organic matter (30). Due to similar chemical properties to nucleic acids, humic compounds are typically co-purified during standard nucleic acid extraction methods; these compounds are established inhibitors of PCR reactions (31), and most other subsequent analyses were performed on DNA (32, 33). Therefore, additional treatment is required to remove humic substances when performing DNA extraction on material that is known to be rich in organic matter. Chemical flocculation, which employs multivalent cations (such as Al^3+^) to bind and remove organic inhibitors, has been demonstrated as an effective treatment for the removal of humic compounds when extracting DNA from environmental samples such as soil (32, 34, 35).

We have previously demonstrated the utility of solid-phase reversible immobilization (SPRI) paramagnetic beads as a low-cost method for the purification of nucleic acids (36). Here, we show that using a pre-treatment with aluminum ammonium sulfate [AlNH_4_(SO4)_2_, also known as alum], combined with mechanical and chemical lyses with bead beating and a guanidinium isothiocyanate (GITC)-based lysis solution, followed by nucleic acid extraction with SPRI beads, we can obtain *M. ulcerans* DNA from possum excreta without comprising diagnostic yield, at 1/6 the cost of commercial reagents. Furthermore, the method was demonstrated to be adaptable for DNA extraction from biological swabs collected from possums with suspected *M. ulcerans* infection and for the extraction of mycobacterial genomic DNA.

## MATERIALS AND METHODS

### Bacterial strains

*M. ulcerans* clinical isolate JKD8049 (26) (or DNA from this isolate) was used as positive control material for all experiments. Additional strains used in this study were as follows: *Mycobacterium abscessus* TPS8830 (unpublished), *Mycobacterium chimera* DMG1600132 (37), *Mycobacterium marinum* “M” strain (38), *Mycobacterium smegmatis* MC2155 (39), *Mycobacterium virginiense* JKD8049 (unpublished), and *Staphylococcus epidermidis* BPH0747 (40).

### Biological specimen collection

Voided possum excreta (*N* = 78) were collected as part of structured possum excreta surveillance studies using the standardized manner described in the study by Vandelanoote et al. (8) that included repeat survey of the Mornington Peninsula (located 70 km south of Melbourne) and Geelong (8), extending to new areas in inner metropolitan Melbourne, collected between 2022 and 2023. Excreta were transported to the laboratory within 24 hours of collection under refrigeration using cold packs and stored at −20°C until the time of processing.

Dry, flocked swabs (Copan 8155C1S) were used to sample cutaneous lesions and, in the case of necropsied possums, organs and/or body cavities (oral, cloaca, pouch) of wild, native possums during active trap-and-release and passive necropsy-based surveillance studies conducted around Melbourne and Geelong, Victoria, between November 2021 and December 2022 (41). Collected swabs were stored in individual tubes at −20°C until the time of processing.

### Preparation of contrived *M. ulcerans*-positive possum excreta samples

Possum excreta spiked with a standardized inoculum of *M. ulcerans* were made as follows: 20 mg of *M. ulcerans* JKD8049 harvested from a Brown and Buckle slope was added to 5 mL of nuclease-free water in a 50-mL centrifuge tube. To create a homogenous suspension, a sterile 1-mL pipette tip was added and the tube was vortexed. Five grams of possum excreta known to be *M. ulcerans* IS*2404* qPCR negative, crushed to the consistency of filter coffee grounds, was added to the culture and pipette tip and then vortexed to mix. The sample was stored at 4°C and allowed to soak overnight before testing.

### DNA extraction buffers, solutions, and paramagnetic beads

The 15 mM AlNH_4_(SO4)_2_ wash solution contained 15 mM AlNH_4_(SO4)_2_×12H_2_O and 100 mM Tris HCl (pH 6.0). The 6M GITC lysis buffer comprised of 6M GITC, 150 mM Tris HCl (pH 8.0), 90 mM dithiothreitol, 30 mM ethylenediaminetetraacetic acid (pH 8.0), and 3% Triton-X 100 (volume/volume) and was stored protected from light. Note that this solution precipitates when stored below 28°C. SPRI on carboxylated paramagnetic beads (SpeedBeed Magnetic Carboxylate Modified Particles 100 mL, Azide 0.05% 65152105050350, GE Healthcare) was functionalized for DNA binding as described in reference (42) and stored at 4°C, protected from light. Unless otherwise specified, chemicals were purchased from Sigma Aldrich.

### Possum excreta DNA extraction

To allow direct comparison of DNA extraction methods, individual excreta pellets were divided into equal halves (determined by weight) using a disposable wooden spatula. One-half was processed with the in-house SPRI-bead method and the other half the DNeasy PowerSoil Pro Kit (Qiagen Cat 47016) as per the manufacturer’s instructions.

For the SPRI-bead method, up to 100 mg of possum excreta crushed to the consistency of filtered coffee grounds was added to a pre-autoclaved (121°C, 15 minutes) screw cap tube containing 1 g of 0.1 mm zirconium oxide beads. A 1-mL aliquot of 15 mM AlNH_4_(SO4)_2_ wash solution was added, the tube was vortexed for 1 minute and then centrifuged at 17,000 × *g* for 1 minute, and 700 µL of the supernatant was discarded. A 700-µL aliquot of 6M GITC lysis solution was added, and the tube was subjected to one cycle of 6,000 rpm for 40 seconds in a bead beater. The tube was centrifuged at 17,000 × *g* for 1 minute, and 600 µL of supernatant was transferred to a new microcentrifuge tube. To the supernatant, 225 µL of 10M ammonium acetate; then, 75 µL of 20% (weight/volume) sodium dodecyl sulfate was added, with inversion of the tube following the addition of each. The tube was centrifuged at 17,000 × *g* for 1 minute, and 300 µL of the supernatant was transferred to a new microcentrifuge tube, and 200 µL of 100% ethanol was added to precipitate the DNA.

For each sample to be processed, an 85-µL aliquot of functionalized DNA-binding SPRI beads was added into a well of a 96-well plate; then, 165 µL of the supernatant/ethanol mix was added to the same well, with gentle mixing by pipetting. After all samples and controls were aliquoted, the 96-well plate was incubated at room temperature for 5 minutes to allow SPRI bead and DNA binding. The plate was then mounted on a magnetic rack until the supernatant was clear (variable time depending on specimens, less than 1 minute for non-possum excreta controls to 5 minutes for contaminated samples), with the DNA-SPRI bead complexes fixed to the bottom of the plate by the magnets. The supernatant was discarded by pipetting, with the plate still mounted on the magnetic rack. To ensure adequate washing of pellets and removal of any trapped debris, the DNA-SPRI bead pellets were resuspended by pipetting in 250 µL of 80% (volume/volume) ethanol with the plate off the magnetic rack. Contaminated pellets discolored the wash solution green/brown and released particulate matter. The plate was remounted on the magnetic rack, and once the DNA-SPRI pellets formed, the wash solution was discarded by pipetting and any visible debris was removed with a pipette tip. A second 80% ethanol wash was performed with the plate off the magnet. The plate was remounted on the magnet to discard the wash. With each wash, the time required for the DNA-SPRI pellet to form and the wash solution to clear decreased. Pellets were air dried at room temperature for 5 minutes (or until matte in appearance, avoiding a cracked pellet, which indicates overdrying and is associated with low DNA yield) with the plate on the magnet. The plate was then removed from the magnetic rack, and DNA was eluted from the SPRI beads with a 1-minute incubation in 30 µL of nuclease-free water. The plate was remounted on the magnetic rack, and the eluate was transferred to a clean 96-well plate and then stored at −20°C if not immediately tested. All steps of the DNA extraction and purification method were performed at room temperature.

### Swabs collected from possums

Swabs were inoculated into 1 mL of phosphate-buffered saline and vortexed for 1 minute; 200-µL aliquots of the sample solution were processed by both the in-house method and the DNeasy Blood & Tissue Kit (Qiagen Cat 69504) per the manufacturers’ instructions.

For the SPRI-bead method, the 200-µL sample aliquot was inoculated directly into a pre-autoclaved, screw cap tube containing 1 g of 0.1 mm zirconium oxide beads and 500 µL of 6M GITC lysis solution. The tube was subjected to one cycle of 6,000 rpm for 40 seconds in a bead beater and then incubated at room temperature for 5 minutes. Centrifugation, protein precipitation with ammonium acetate and sodium dodecyl sulfate, repeat centrifugation, and DNA precipitation steps were performed as outlined in the method for DNA extraction from possum excreta. To the 500 µL DNA/ethanol mix in a 1.5-mL microcentrifuge tube, 255 µL of functionalized DNA-binding SPRI beads was added and mixed by gentle pipetting. The tube was incubated for 10 minutes at room temperature and then placed in a 1.5-mL microcentrifuge magnetic rack until the supernatant was clear. The following steps were performed with the tube mounted in the magnetic rack without removal: the supernatant was discarded; the DNA-SPRI bead pellet was washed twice (without resuspending) with 1.5 mL of 80% (volume/volume) ethanol, and the supernatant was discarded after each wash; the pellet was airdried at room temperature for 5 minutes. The tube was then removed from the magnetic rack and DNA eluted from the SPRI beads with a 1-minute incubation in 100 µL of nuclease-free water. The tube was remounted in the magnetic rack, and the eluate was transferred to a clean 1.5-mL microcentrifuge tube and then stored at −20°C if not immediately tested.

### DNA extraction from mycobacterial culture

A heaped 10-µL loop of JKD8049 *M. ulcerans* harvested from a Brown and Buckle slope was inoculated directly into a pre-autoclaved, screw cap tube containing 1 g of 0.1 mm zirconium oxide beads and 700 µL of 6M GITC lysis solution. The sample was processed as outlined in the method for swabs collected from possums, from the bead beating step, including centrifugation, protein precipitation, and repeat centrifugation up to DNA precipitation with ethanol, when 600 µL of supernatant was transferred to a new microcentrifuge tube; then, 400 µL of 100% ethanol was added, and the tube was inverted to mix. One milliliter of the supernatant/ethanol mix was evenly divided between two microcentrifuge tubes, and 255 µL of functionalized DNA-binding SPRI beads was added to each tube. Tubes were incubated for 10 minutes at room temperature and then placed in a 1.5-mL microcentrifuge magnetic rack until supernatants were clear. The remaining extraction process of ethanol washes and DNA elution in 1.5-mL tubes were as outlined for the possum swab extraction; except following elution in 100 µL of nuclease-free water, both eluates were pooled together in a clean microcentrifuge tube and stored at −20°C until being required for testing. Genomic DNA was quantified using a fluorometer (Qubit 4 Fluorometer, Thermo Fisher), and the quality was assessed with the 4200 TapeStation System (Agilent).

### IS2404 TaqMan quantitative PCR

The primers targeting the IS*2404* insertion sequence in *M. ulcerans* were as described previously (20) (oligonucleotides purchased from Integrated DNA technologies), with TaqMan Exogenous Internal Positive Control Reagents—VIC Probe (Applied Biosystems Cat # 4308323) and SensiFAST probe No-ROX Kit (Bioline Cat # BIO-86005). Each 20-µL reaction consisted of the following: 2× SensiFAST Probe No-ROX mix, 0.4 µM IS*2404* TF, 0.4 µM IS*2404* TR, 0.1 µM IS*2404* TP, 10× TaqMan Exo IPC Mix, 50× TaqMan Exo IPC DNA, 3.2 µL of nuclease free water, and 2 µL of sample DNA. No-template controls, *M. ulcerans* genomic DNA-positive control, and TaqMan internal-positive control block (indicates if PCR inhibition has occurred) were included in every qPCR run. Amplification and detection were performed on the QuantStudio 1 (Thermo Fisher) platform using the following program: 95°C for 5 minutes, then 45 cycles of 95°C for 10 seconds and 60°C for 20 seconds. A cycle threshold (Ct) ≤ 40 was interpreted as positive for the presence of *M. ulcerans*. This cut-off was in accordance with previous performance evaluations of the assay (8, 43). Run analyses were performed using Design & Analysis Software v2.5.0 (Thermo Fisher Scientific).

### Statistical analyses

Data comparisons were performed using GraphPad Prism (v10.2.0). Correlation analyses were performed using Spearman’s rank correlation coefficient to compare the performance of DNA extraction methods using IS*2404* qPCR Ct values (continuous variable) as the measured comparator. The Wilcoxon matched-pairs signed rank test with a two-tailed *P* value (*P* < 0.01) was used to test the null hypothesis (no difference between mean IS*2404* Ct values).

## RESULTS

### Development of a SPRI-bead DNA extraction and purification method for possum excreta specimens

**Fig 1:**
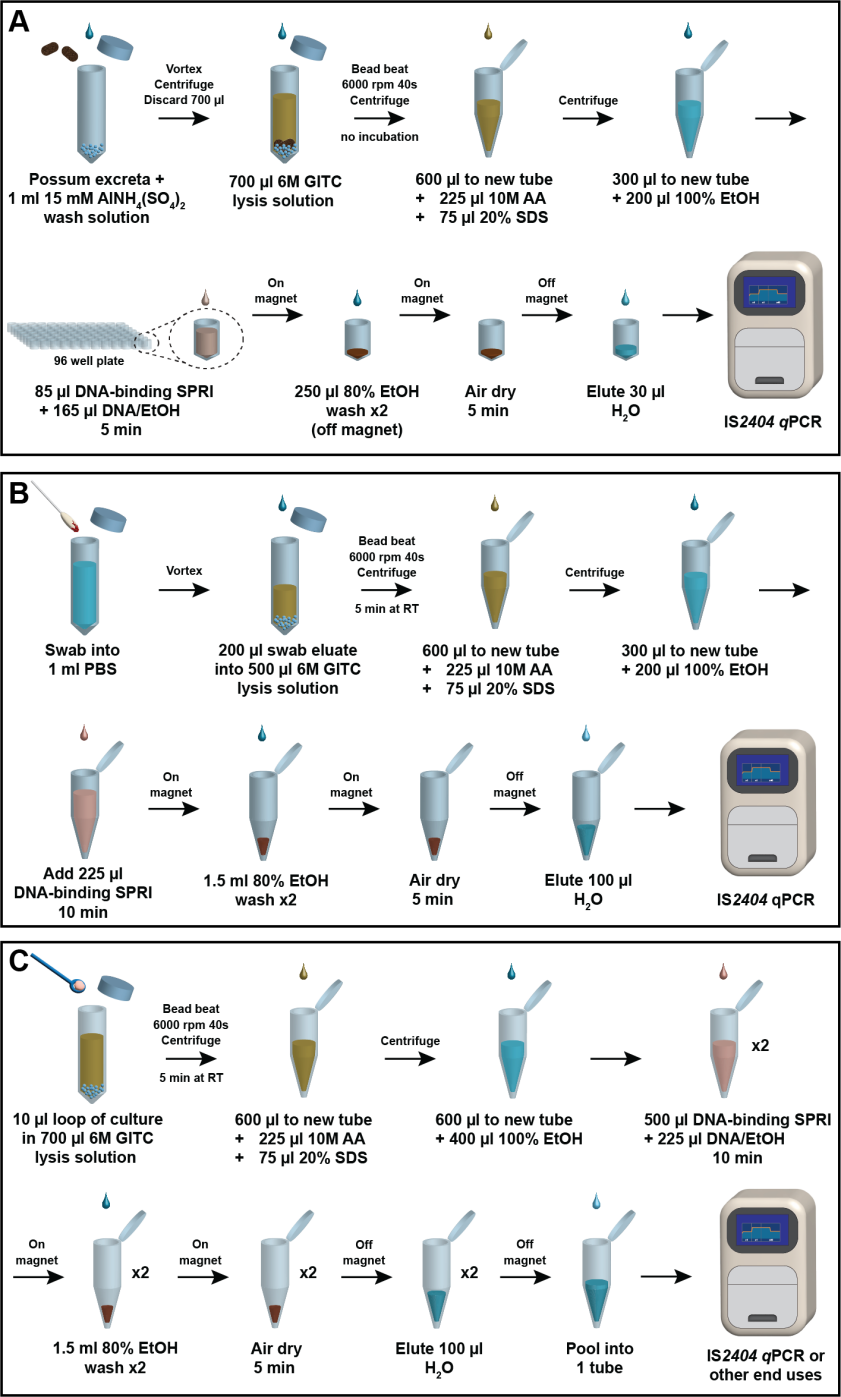
Overview of SPRI-bead-based DNA extraction and purification methods developed for (**A**) possum excreta specimens; (**B**) biological swabs taken from possums with suspected *M. ulcerans* infection; (**C**) pure mycobacterial culture. All steps are performed at room temperature. GITC, guanidinium isothiocyanate; AA, ammonium acetate; SDS, sodium dodecyl sulfate; EtOH, ethanol; IS*2404*, insertion sequence *2404*; qPCR, quantitative polymerase chain reaction; PBS, phosphate-buffered saline; RT, room temperature.

An overview of the method developed is shown in Fig. 1A. To reduce the quantity of humic compounds contaminating the final DNA extraction, the following measures were employed: inclusion of a 15-mM AlNH_4_(SO4)_2_ (pH 6.0) prewash, crushing of possum excreta samples, limiting the time allowed for ongoing chemical lysis after mechanical lysis, resuspension of the DNA-SPRI pellet off the magnet during 80% ethanol wash steps, and removal of any residual debris. Each of these measures was found to improve the clarity of the DNA extraction and/or reduce PCR inhibition when the DNA was tested by IS*2404* qPCR.

### *Mycobacterium ulcerans* is stable in possum fecal material

**Fig 2:**
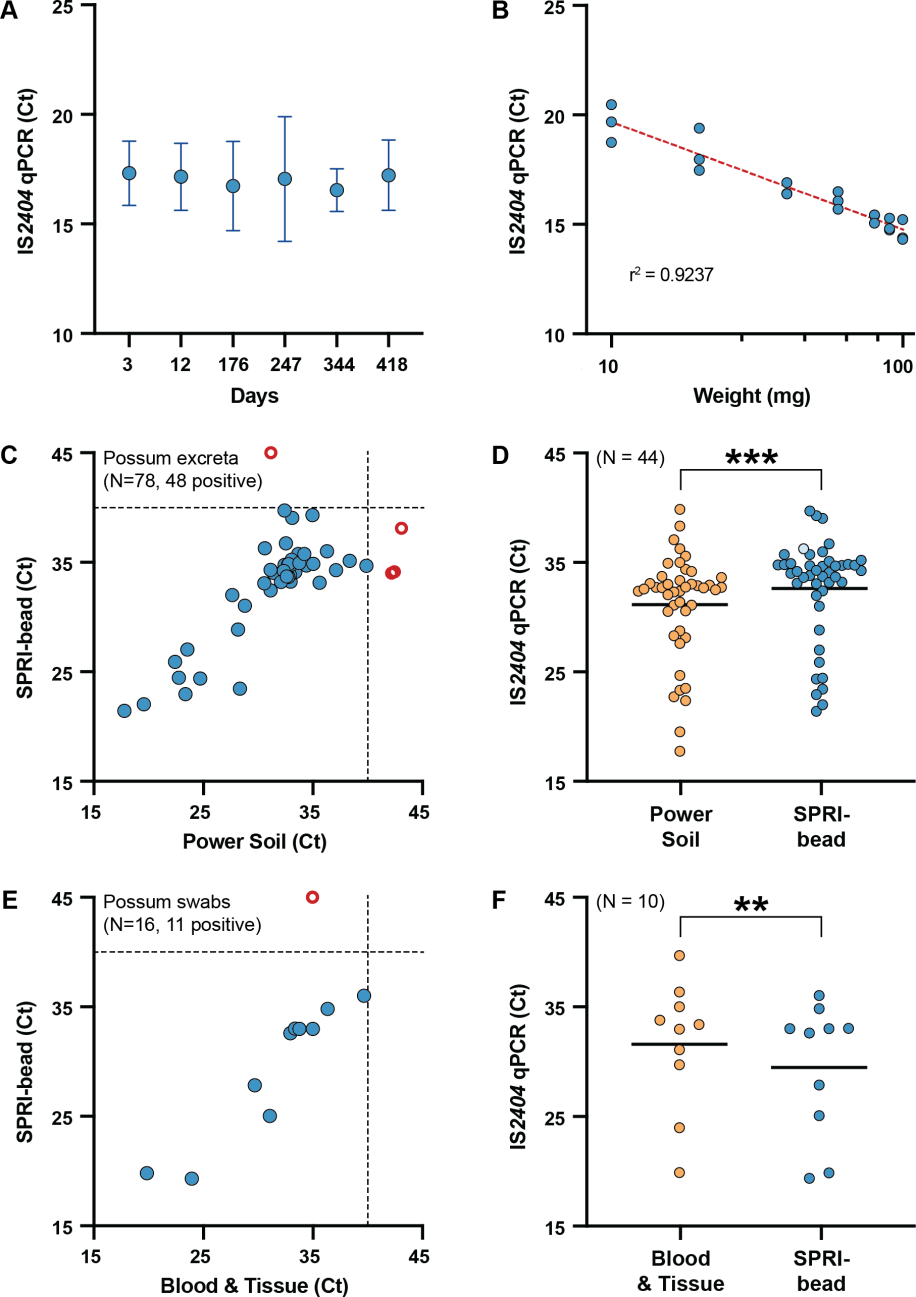
Development and validation of SPRI-bead-based DNA extraction methods for detecting *M. ulcerans*. (**A**) Stability profile of a contrived *M. ulcerans*-positive possum excreta. DNA extractions of 50 mg of contrived positive sample were performed in triplicate (mean value plotted) at each time interval and tested for the presence of *M. ulcerans* insertion sequence (IS) *2404* using qPCR. Error bars represent the 95% CI (**B**). Efficiency of SPRI-bead-based DNA extractions of increasing weights of contrived positive possum excreta, as assessed by IS*2404* qPCR. DNA extractions performed in biological triplicate are shown. Dotted red line shows the square of the Spearman’s correlation coefficient. (**C**) Comparison of Qiagen PowerSoil Pro and a SPRI-bead-based method for DNA extraction from possum excreta. Possum excreta samples collected during surveillance studies were halved and processed by the “gold-standard” PowerSoil Pro Kit and SPRI-bead method. Blue circles show 44 samples with DNA extractions that tested IS*2404* positive by both methods. Dotted lines indicate the Ct ≤ 40 cut-off for the IS*2404* qPCR, above which samples are interpreted as negative. Open red circles indicate samples not detected by one method. The 30 samples that tested negative by both DNA extraction methods are not shown. (**D**) Comparison of Ct values for the 44 possum excreta DNA extractions that tested IS*2404* qPCR positive by both methods. The single pale blue circle represents a SPRI bead-extracted sample that was PCR inhibited on initial testing but tested positive after dilution of the DNA extract. Black horizontal bars represent the mean IS*2404* qPCR Ct value for each method. The null hypothesis (no difference between means) was rejected for *P* < 0.01 (Wilcoxon matched-pairs signed rank test, two-tailed *P* value). ***P = 0.0002. (**E**) Comparison of the Qiagen Blood & Tissue Kit and a SPRI-bead-based method for DNA extraction from possum tissue swabs. Swabs collected from possums were each eluted in PBS, and equal aliquots of eluate were DNA extracted using either the Blood & Tissue Kit or SPRI-bead method. Blue circles show samples with DNA extractions that tested IS*2404* positive by both methods. The five samples that tested negative by both DNA extraction methods are not shown. (**F**) Comparison of the 10 possum swab samples that tested positive by both DNA extraction methods. Black horizontal bars represent the mean IS*2404* qPCR Ct value for each DNA extraction method. The null hypothesis (no difference between means) was rejected for *P* < 0.01 (Wilcoxon matched-pairs signed rank test, two-tailed *P* value). **P = 0.0020.

To facilitate development and optimization of the SPRI-bead DNA extraction method, pooled possum excreta known to be IS*2404* qPCR negative was spiked with a standardized inoculum of *M. ulcerans*. Interval testing of 50 mg of this spiked possum excreta, in triplicate, over 418 days indicated that the concentration of *M. ulcerans* remained stable in this matrix (stored in a sealed 50-mL tube at 4°C) over this period, as reflected in unchanged IS*2404* qPCR Ct values (Fig. 2A). This pool of stable, spiked excreta was used to create a series of positive possum excreta specimens from 10, 20, 40, 60, 80, 90, to 100 mg for subsequent SPRI-bead assay performance evaluations.

For quality control, the stability of the 6M GITC lysis solution and the functionalized DNA-binding SPRI beads when used in our assay were also determined. Batch testing of 6M GITC lysis solution of various ages demonstrated consistent performance for at least 1,094 days (2 years, 11 months, 29 days; Fig. S1A). No difference was noted if the solution was stored protected from light at room temperature and then warmed until the precipitated GITC dissolved into solution or if the 6M GITC buffer was stored protected from light at 28°C throughout. Similarly, batch testing of the DNA-binding SPRI beads was performed consistently for at least 1,008 days (2 years, 9 months, 2 days; Fig. S1B), indicating no deterioration of these solutions over at least 2.8 years.

### Performance of SPRI bead DNA extraction for possum excreta

DNA was extracted by the SPRI-bead method from triplicate preparations of the seven increasing weights of the *M. ulcerans*-spiked excreta. The resulting DNA preparations were then tested for the presence of *M. ulcerans* by IS*2404* TaqMan qPCR (20). We observed a consistent, linear reduction in Ct as increasing weights of possum excreta were tested, up to 100 mg (Fig. 2B). Above 100 mg, PCR inhibition was observed, as monitored by the presence or absence of the qPCR internal positive control (Table S1). This observed decrease in performance when processing above 100 mg of excreta was likely due to the higher quantity of humic acids and other interferents present in possum excreta that were not effectively removed during extraction.

### Comparison of SPRI bead DNA extraction with the DNeasy PowerSoil Pro Kit for possum excreta

Having established the maximum volume of material the SPRI-bead DNA extraction method could process, we sought to further validate the protocol by screening 78 possum excreta specimens from a previous study we knew to have originated from sites with a high probability of harboring *M. ulcerans* (8). Individual excreta pellets were divided into equal halves (determined by weight), with half processed using our SPRI-bead method and the other half the DNeasy PowerSoil Pro Kit (considered the “gold standard”). For large brushtail possum excreta pellets which could exceed 300 mg in weight, 50 mg of the excreta was tested by each extraction method. The weights of possum excreta tested ranged from 21 mg to 96 mg, with an average weight of 53 mg (95% CI 50–56 mg) (Fig. S2). The resulting DNA preparations were tested for the presence of *M. ulcerans* using the IS*2404* qPCR. A comparison of the efficacy of the two DNA extraction methods when processing the paired possum excreta samples is shown in Fig. 2C and D. Of the 78 possum excreta samples extracted, 44 tested IS*2404* qPCR positive by both methods and 30 tested IS*2404* qPCR negative by both methods, while 4 samples were detected by only one method (Fig. 2C; Table S2).

Three samples had discordant IS*2404* qPCR results, testing positive when extracted with the SPRI-bead method with Ct values ranging from 34 to 38, but negative when extracted by the gold standard method with corresponding sample Ct values of 42–43. These three samples were interpreted as negative due to being above the positive interpretation cut-off for the IS*2404* qPCR of Ct ≤ 40. PowerSoil Pro extractions of additional possum excreta collected from the same locations at the same time as each of the three discordant samples had IS*2404* Ct values of 32–34, suggesting these were likely true positive samples. Two samples that were IS*2404* positive when extracted by the gold-standard method initially yielded “no result” following SPRI bead extraction due to PCR inhibition. Testing 1/2 and 1/4 dilutions of the SPRI bead DNA extracts enabled one of these samples to be detected at both dilutions (1/2 dilution: Ct 37.69, 1/4 dilution: Ct 36.30); however, the other sample remained inhibited at both dilutions and was interpreted as a false negative result. This PCR-inhibited sample was 96 mg in weight. Based on the above, there were 48 true positive and 30 true negative samples. Therefore, our in-house DNA extraction method had a sensitivity of 97.9%, specificity of 100.0%, positive predictive value of 100.0%, and negative predictive value of 96.77%.

For the 44 samples from which *M. ulcerans* was detected in the DNA extractions processed by both methods, there was overall good concordance between the two methods (Spearman *r* = 0.6947, *P* < 0.0001). However, the mean IS*2404* Ct of 32.65 obtained with the SPRI-bead method was significantly higher than the PowerSoil Pro mean Ct of 31.18 (*P* = 0.0002) (Fig. 2D). This mean difference of 1.47 qPCR cycles suggested an approximately threefold decrease in sensitivity of the SPRI-bead method compared with the PowerSoil Pro Kit.

### Comparison of SPRI bead DNA extraction with the DNeasy Blood & Tissue Kit for swabs taken from possums

We also explored the suitability of the SPRI-bead method for detecting *M. ulcerans* DNA from swabs collected from live or necropsied possums, some with ulcerated lesions suspicious of *M. ulcerans* infection. A total of 16 swabs, collected from 10 possums, were available for testing (see Table S3 for details of possum specimens). Swab material was eluted in PBS before equal volumes of the eluate were processed for DNA extraction using the DNeasy Blood & Tissue Kit (considered the “gold standard” for comparison purposes) and a SPRI-bead method omitting the alum wash step (outlined in Fig. 1B). The DNA extraction from each method was then tested for the presence of *M. ulcerans* by IS*2404* qPCR. Using the DNeasy Blood & Tissue Kit, 11 swab eluates tested positive and 5 were negative, compared with 10 positive and 6 negative swab eluates using the SPRI-bead method. The single false negative result from the SPRI-bead method corresponded with an eluate that had been stored for >300 days before testing by this method and had a relatively high Ct value of 35; therefore, this specimen may have degraded during storage beyond the limit-of-detection for the IS*2404* qPCR. Based on this small sample size, the sensitivity of the SPRI-based method was 90.9%, specificity 100.0%, positive predictive value 100.0%, and negative predictive value 83.3%.

Similar to the possum excreta DNA extracts, there was good concordance between the two methods (Spearman *r* = 0.9362, *P* < 0.0001; Fig. 2E). However, in this comparison, the SPRI-bead method was observed to have a significantly lower mean Ct of 29.44 than the mean Ct of 31.56 for the Blood & Tissue Kit (*P* = 0.0020; Fig. 2F). The mean difference of 2.12 PCR cycles indicated the SPRI-bead method was approximately fourfold more sensitive than the gold-standard method.

### SPRI bead DNA extraction for mycobacterial cultures

We also adapted the SPRI-bead method for preparation of genomic DNA from pure cultures of *M. ulcerans* and other mycobacteria (Fig. 1C). The method omitted the alum wash step since humic substances are not present in pure bacterial culture, and an increased volume of lysate was processed (1,000 µL compared with 165 µL) to maximize DNA yield. Three genomic extractions of *M. ulcerans* yielded an average of 24.2 µg (range 20.8–29.6 µg) of DNA with a peak size of 8,865 bp (range 6,822 to 10,314 bp). Similarly high amounts of genomic DNA were also obtained from *Mycobacterium abscessus* (23.3 µg; 10,336 bp), *Mycobacterium chimera* (14.5 µg; 10,910 bp), *Mycobacterium marinum* (26.8 µg; 12,586 bp), *Mycobacterium smegmatis* (28.3 µg; 10,657 bp), and *Mycobacterium virginiense* (28.4 µg; 13,619 bp). The full quality control metrics of these mycobacterial genomic DNA extractions and two comparators, including a chloroform extraction of *M. ulcerans* (10,462 bp) (44) and a high-quality *Staphylococcus epidermidis* extraction performed using the Qiagen Blood & Tissue Kit with pre-treatment to weaken the Gram-positive cell wall (45) that was subsequently PacBio sequenced (40) (56,896 bp), is shown in Fig. S3.

### Cost comparison of DNA extraction methods

We assessed the material costs needed to prepare DNA from 96 excreta samples by both our SPRI-bead method and the Qiagen PowerSoil Pro Kit. The cost per sample for the PowerSoil Pro method depended on the kit size, with a kit of four 96-well plates costing AUD $8 per sample, increasing to AUD $12 per sample for the smaller, 50-tube kit. By comparison, our SPRI-bead method cost AUD $2 per sample irrespective of 384 or 50 samples being processed. Similarly, the cost for processing biological swabs collected from possums using the Qiagen Blood & Tissue Kit was AUD $10 per sample for a 50-tube kit, whereas our SPRI-bead method was only AUD $2. See Table S4 for the full cost breakdown of SPRI-bead methods.

## DISCUSSION

Extensive environmental surveillance studies have been the foundation of field-based research leading to the discovery that BU is a zoonosis (8–12). Possum excreta surveys have been instrumental in ascertaining transmission pathways and predicting the geographic risk of BU spread to humans in Victoria, Australia (8, 10). Molecular analytical methods based on IS*2404* qPCR have been the mainstay for these surveys, but the high sensitivity and specificity of this assay (20) are countered by the relatively high cost of the commercial nucleic acid extraction reagents needed. For environmental surveillance projects involving the collection of thousands of excreta specimens, research budgets can be quickly exhausted, hindering endeavors to halt disease spread through large-scale measure-intervene-measure public health responses. Here, we addressed this bottleneck by developing lower-cost nucleic extraction methods.

Our possum excreta DNA extraction method (Fig. 1A) utilized AlNH_4_(SO4)_2_ as a chemical flocculant to reduce the quantity of humic compounds present in the final DNA extraction that would otherwise interfere with subsequent qPCR analyses. To ensure that older possum excreta samples solidified from prolonged environmental exposure would adequately absorb the 15-mM AlNH_4_(SO4)_2_ (pH 6.0) wash solution, samples were crushed to a fine-ground coffee consistency. At pH 6.0, Al^3+^ complexes with humic substances and precipitates (35, 46). After centrifugation, the washed pellet was incubated with the 6M GITC lysis solution (pH 8.0) and subjected to bead beating to ensure efficient mycobacterial cell wall disruption (47). This change in pH to 8.0 precipitates superfluous Al^3+^ preventing unwanted complexing of DNA (32, 35) released during bead beating. No incubation in the 6M GITC lysis solution was specified in this protocol; the time required to perform bead beating, subsequent centrifugation, and transfer of supernatant to a new tube allowed adequate chemical lysis. Furthermore, digestion of possum excreta in the 6M GITC lysis solution for more than 45 minutes prior to protein precipitation should be avoided as it results in the release of excessive humic substances and subsequent PCR inhibition. Extended digestion may be an issue when processing many samples. The lysate was further clarified with the addition of ammonium acetate and sodium dodecyl sulfate to precipitate proteinaceous materials. After centrifugation, one-third of the resulting lysate was mixed with ethanol to precipitate the DNA, and approximately one-third of the DNA/ethanol mix was transferred to a 96-well plate containing SPRI beads, for a final SPRI:DNA ratio of 0.5. While the IS*2404* element is around 1,300 bp in size (20, 25) and the product of the IS*2404* qPCR used is only 59 bp (20), the original study validating the IS*2404* PCR method used 515-bp amplicons as a positive control (20). Therefore, our protocol used a DNA:SPRI ratio of 0.5 to target binding of DNA fragments approximately 600 bp in size. Finally, the DNA-SPRI bead complex was immobilized using a magnet and washed with ethanol twice to remove impurities, and then, the DNA was eluted from the beads using a small volume (30 µL) of nuclease-free water.

When adapting this method to extract DNA from swabs collected from possums (Fig. 1B) and mycobacterial cultures (Fig. 1C), the alum wash step was omitted since neither of these sample types contained humic substances. A 5-minute incubation in the 6M GITC lysis solution was included for enhanced chemical lysis of the mycobacterial cell wall following mechanical disruption with the bead beater, since no additional humic substances would be released. The increased volume of sample lysate processed in both methods required a corresponding increase in the volume of SPRI beads added. Therefore, the incubation time of the SPRI beads with precipitated DNA was doubled to 10 minutes to ensure maximal DNA binding. Since contamination of the DNA-SPRI pellet with humic substances and debris was not a concern for either swabs collected from possums or pure mycobacterial cultures, the ethanol wash steps were performed on the magnet without resuspension in both protocols.

Integral to all three of our described nucleic acid extraction methods were the 6M GITC lysis solution and functionalized DNA-binding SPRI beads. When stored appropriately, we have demonstrated both reagents to perform consistently for at least 2.8 years, indicating they are amenable to bulk preparation for later use, removing the onus of preparing multiple solutions immediately prior to testing. Furthermore, use of in-house solutions overcomes supply chain issues present with commercial reagents and kits, such as the global dearth of nucleic acid extractions kits experienced during the SARS-CoV-2 pandemic (48, 49).

In view of the large number of possum excreta routinely processed in *M. ulcerans* surveillance studies, we prioritized a protocol amenable to high-throughput processing of samples in a 96-well plate format rather than extraction of the maximal volume of lysate in individual tubes. Therefore, we downscaled our method to process 1/9 of the lysed possum excreta sample (300 µL of 900 µL lysate post protein precipitation, then 165 µL of the 500 µL of precipitated DNA), as compared with the PowerSoil Pro Kit, which used the full lysate volume. Despite the much lower fraction of sample extracted, paired comparison of extractions indicated our SPRI-bead method was only approximately threefold less sensitive than the commercial kit, with overall good concordance in results (*r* = 0.6947, *P* < 0.0001) and a calculated sensitivity of 97.9% and specificity of 100.0% for the 78 possum excreta samples tested.

Discordance between the two methods for three of the possum excreta samples may have been due to unequal distribution of *M. ulcerans* within the excreta specimens, resulting in failure of the PowerSoil Pro method to detect the known positive samples. Of note, the single SPRI bead-extracted sample that failed to yield a result due to PCR inhibition corresponded with the heaviest surveillance specimen tested (96 mg) that was close to the 100-mg maximum extractable weight determined when developing the method based on contrived *M. ulcerans*-spiked possum excreta. Due to dietary differences between individual possums, the humic substance content of excreta samples can vary. This PCR-inhibited result suggested that for some possum excreta samples, even 100 mg may contain humic compounds that exceed the flocculation capacity of the 15 mM AlNH_4_(SO4)_2_ wash; therefore, extraction of a lower weight of these samples is recommended if dilution of the DNA extraction is insufficient to overcome PCR inhibition. It could also be argued that routine use of a lower sample input may be prudent to avoid PCR inhibition, without necessarily incurring a high number of false negative results. Indeed, the average weight of the possum excreta samples tested by each method in the experiment was only 53 mg. Therefore, we recommend using between 50 and 90 mg of possum excreta when performing DNA extraction with our SPRI-bead method.

Since fewer possum swabs are collected during surveillance studies compared with possum excreta samples, for this DNA extraction method, we developed a tube-based method in which 1/3 of the lysed sample was extracted. When combined with DNA-binding SPRI beads at a ratio of 0.5, the final volume fits into a single 1.5-mL tube and formed a DNA-SPRI pellet that did not take excessive time to bind to a magnet or air dry. Although pairwise comparison of the possum swab samples demonstrated our SPRI-bead method was superior, with an estimated fourfold sensitivity above the commercial Blood & Tissue Kit, calculation of the overall sensitivity of the method was hindered by the small experimental sample size of 16 swabs and availability of only retrospective samples for testing (around which our method was developed). Since only 11 true positive samples were tested, the single false negative resulted in a calculated sensitivity of only 90.9%, with a specificity of 100.0%. Of note, this false negative was from a sample with a relatively high a Ct of 35 when tested by the gold standard Blood & Tissue Kit that had been stored for over 300 days prior to extraction with the SPRI-bead method; therefore, this specimen may have degraded during storage beyond the limit-of-detection for the IS*2404* qPCR. The sensitivity testing of our method was disadvantaged by being limited to retrospective testing of existing samples that were eluted in 1 mL of PBS and then stored at −20°C for months after an aliquot was tested by the gold-standard method. Ideally, if prospective testing were performed, swabs would be resuspended directly in the 6M GITC solution immediately after collection, removing dilution of the sample in PBS and deterioration of DNA over time and from repeated freeze thawing.

Our SPRI-bead-based methods yielded DNA extractions from environmental samples, diagnostic swabs, and pure mycobacterial cultures that were suitable for end use in IS*2404* PCR reactions. To functionalize the DNA-binding SPRI beads, we used the method described by Jolivet & Foley, which was developed as a low-cost substitute for AMPureXP (Beckman Coulter) beads, designed for DNA clean-up (42). Our protocol used a SPRI:DNA ratio of 0.5 to target approximately 600 bp, rather than the 1.8 times ratio typically recommended by most commercially available SPRI bead-based DNA clean-up reagents that target a peak of 200 bp, optimized for PCR and next-generation sequencing (50). This partially accounts for the relatively low average peak DNA size of our mycobacterial genomic DNA extractions (10,588 bp), as compared with the commercial kit extraction of *S. epidermidis* (56,896 bp). While bead beating can result in DNA shearing, the upper size of >60,000 bp for the *M. marinum* and 52,397 bp for the *M. virginiense* genomic extractions using our SPRI-bead method indicates the method is capable of extracting long-length DNA fragments. However, if DNA is specifically intended for long-read sequencing, the use of SPRI beads functionalized for high-molecular weight DNA selection [through alteration of polyethylene glycol 8000 and NaCl concentrations (51)] or a commercial product like SPRIselect (Beckman Coulter) would be recommended instead, with inclusion of an RNA depletion step.

Interspecies differences also contribute to the lower average peak DNA size for our mycobacterial genomic extractions compared with the *S. epidermidis* isolate. The mycobacterial cell wall is rich in lipids and polysaccharides forming a complex structure that renders the bacterium resistant to lysis, hindering most conventional DNA extraction methods (52, 53); therefore, most protocols developed for mycobacteria are laborious (potentially taking days), with many requiring phenol or phenol/chloroform (53). A chloroform-based DNA extraction method (44) included as a comparator, used for the genomic DNA extraction of *M. ulcerans* JKD6049, had an average peak DNA size of only 10,462 bp, highlighting the difficulties in obtaining high-quality, long-length mycobacterial DNA.

Here, we present low-cost, SPRI-bead-based methods for the extraction of *M. ulcerans* from Australian possum excreta and swabs from possums with suspected *M. ulcerans* infection, as well as the extraction of mycobacterial genomic DNA from pure cultures, which demonstrate comparable performance to commercially available kits, at around 1/6 to 1/5 the cost. Although developed for the IS*2404* qPCR detection of *M. ulcerans* from possum excreta, swabs from possums, and pure bacterial culture, the described methods should be adaptable for the testing of *M. ulcerans* from other environmental sources and swabs from animals other than possums, as well as other bacteria of interest when paired with an appropriate diagnostic PCR. While not implemented in this study, we have previously demonstrated SPRI bead-based nucleic acid extractions to be amenable to high-throughput, low-cost, automation in a 96-well format (36), which could be an area for future assay development.

Collectively, over 4,700 possum excreta samples were tested in the four Victorian *M. ulcerans* surveillance studies discussed in the introduction (8, 9, 11, 12). The 2023 study by Vandelannoote et al. contributed 2,282 samples (8), with over AUD $20,000 spent on commercial DNA extraction kits for this single study. When performed on such scale, the expenditure required for sample processing can be prohibitive to ongoing surveillance when reliant on high-cost commercial reagents. A means to circumvent this barrier prompted this study and, as demonstrated, has been achieved.

The cumulative knowledge from two decades of *M. ulcerans* environmental surveys in Victoria now enables us to predict areas in which humans are at increased risk of disease acquisition and provides local government with a tangible means of interrupting transmission pathways through targeted mosquito control programs (54). However, successful implementation of such a program requires ongoing possum excreta surveillance to inform prediction models and is subject to resourcing and budgetary constraints. By providing an economical means to continue *M. ulcerans* environmental surveillance surveys, this study aims to facilitate efforts to halt and reverse the spread of human BU disease in Victoria. Should these endeavors prove successful, they will also provide vital insights into means to disrupt the spread of BU in other endemic regions in the world.

## SUPPLEMENTAL MATERIAL

Supplemental material aem.01021-24-s0001.pdf Figures S1 to S3 and Tables S1 to S3.

Table S4 aem.01021-24-s0002.xlsx Cost comparisons.

ASM does not own the copyrights to Supplemental Material that may be linked to, or accessed through, an article. The authors have granted ASM a non-exclusive, world-wide license to publish the Supplemental Material files. Please contact the corresponding author directly for reuse.
