# Supplementary material for: A low-cost and versatile paramagnetic bead DNA extraction method for Mycobacterium ulcerans environmental surveillance
Source: Appl Environ Microbiol. 2024 Sep 10;90(10):e01021-24. doi: 10.1128/aem.01021-24 (PMC11497799; doi:10.1128/aem.01021-24)
Supplement: Supplemental material — Figures S1 to S3 and Tables S1 to S3. [file aem.01021-24-s0001.pdf]

SUPPLEMENTARY DATA

A low-cost and versatile paramagnetic bead DNA extraction method for *Mycobacterium ulcerans* environmental surveillance.

Jean Y. H. Lee, Jessica L. Porter, Emma C. Hobbs, Pam Whiteley, Andrew H. Buultjens and Timothy P. Stinear

Contents:

Supplementary Figure 1. Quality control for 6M guanidinium thiocyanate (GITC) lysis solution and functionalised DNA-binding solid-phase reversible immobilisation (SPRI) beads

Supplementary Figure 2. Weights of possum excreta tested for validation of a SPRI-bead based DNA extraction method.

Supplementary Figure 3. Comparison of DNA extractions from pure mycobacterial cultures using SPRI-bead based method.

Supplementary Table 1. Weight series of contrived M. ulcerans-positive possum excreta.

Supplementary Table 2. Collection site and IS*2404* qPCR cycle threshold (Ct) values for possum excreta samples.

Supplementary Table 3. Comparison of the efficiency of a SPRI-bead method with the DNeasy Blood & Tissue kit for extracting *M. ulcerans* DNA from swabs sampled from Australian possums as determined by IS*2404* qPCR.

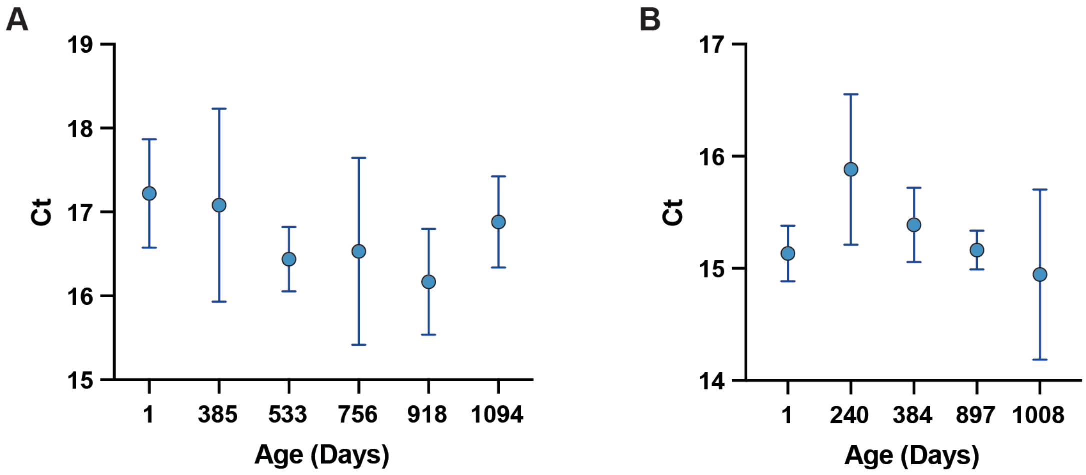

Supplementary Figure 1. Quality control for 6M guanidinium thiocyanate (GITC) lysis solution and functionalised DNA-binding solid-phase reversible immobilisation (SPRI) beads. A. Batches of 6M GITC lysis solution of varying ages were used in the extraction of 50 mg of contrived positive, *M. ulcerans* containing possum excreta, the resultant DNA extraction was then assayed with a TaqMan quantitative polymerase chain reaction (qPCR) targeting insertion sequence (IS) *2404*. Each batch was tested in triplicate. Testing of all batches was performed concurrently. Plotted points represent the mean for each replicate, error bars indicate the 95% confidence interval. There was no significant difference between the lysis ability of each batch [Null hypothesis (no difference between means) was accepted for $p<0.05$. Assessed using one-way analysis of variance (ANOVA) with Tukey's test for multiple comparisons]. **B.** Batches of DNA-binding SPRI beads of varying ages were used in the extraction of 50 mg of contrived positive, *M. ulcerans* containing possum excreta, the resultant DNA extraction was then assayed with IS*2404* qPCR. Each batch was tested in triplicate. Testing of all batches was performed concurrently. Plotted points represent the mean for each replicate, error bars indicate the 95% confidence interval. There was no significant difference in between the DNA-binding of each batch [Null hypothesis (no difference between means) was accepted for $p<0.05$. Assessed using one-way ANOVA with Turkey's test for multiple comparisons].

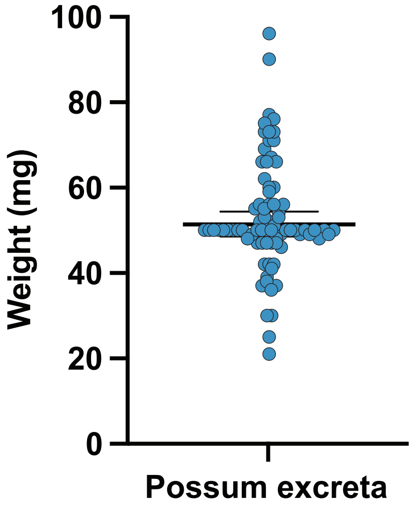

Supplementary Figure 2. Weights of possum excreta tested for validation of a SPRI-bead based DNA extraction method. Seventy-eight possum excreta surveillance samples were divided into two equal halves (by weight) with one half processed by the 'gold standard' PowerSoil Pro kit, the other by our SPRI-bead method. For large excreta pellets, where half the pellet would exceed the predicated 100 mg maximum input for the SPRI-bead method, 50 mg of the pellet was tested by each extraction method. Each circle represents the weight of excreta processed by both DNA extraction methods, for each of 78 samples. Thick horizontal line represents the median weight, error bars indicate the 95% confidence interval.

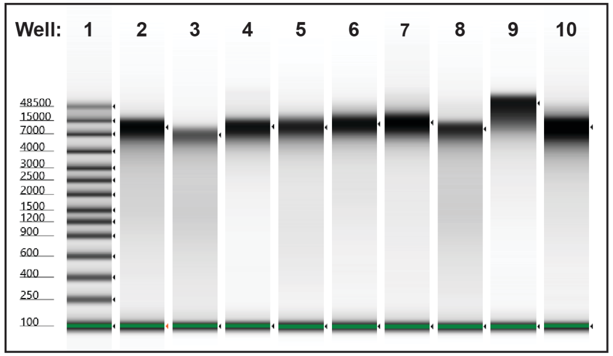

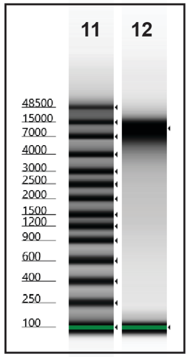

Well	Organism	Strain	Extraction method	Peak (bp)	% Integrated area	From (bp)	To (bp)	Isolate Reference
2	*M. ulcerans*	JKD8049	SPRI-bead	10,314	96.57	3,808	25,279	(1)
3	*M. ulcerans*	JKD8049	SPRI-bead	6,822	92.33	3,553	16,669	(1)
8	*M. ulcerans*	JKD8049	SPRI-bead	9,458	97.32	3,862	22,130	(1)
5	*M. abscessus*	TPS8830	SPRI-bead	10,336	96.15	3,940	30,295	Unpublished
12	*M. chimera*	DMG1600132	SPRI-bead	10,910	91,59	4,236	28,628	(2)
6	*M. marinum*	"M" strain	SPRI-bead	12,586<br>>60,000	87.03<br>0.89	4,865<br>>60,000	54,651<br>>60,000	(3)
4	*M. smegmatis*	MC2155	SPRI-bead	10,657	96.77	4,087	25,549	(4)
7	*M. virginiense*	TPS8833	SPRI-bead	13,619	96.25	4,850	52,397	Unpublished
10	*M. ulcerans*	JKD8049	Chloroform extraction	10,462	99.30	3,920	37,734	(1)
9	*S. epidermidis*	BPH0747	Qiagen Blood & Tissue kit	56,896	99.01	6,063	>60,000	(5)

Supplementary Figure 3. Comparison of DNA extractions from pure mycobacterial cultures using SPRI-bead based method. Note: non-standardised quantity of DNA loaded per well.

Supplementary Table 1. Weight series of contrived *M. ulcerans*-positive possum excreta. A weight series of contrived *M. ulcerans*-positive possum excreta was DNA extracted using a solid-phase reversible immobilisation (SPRI)-bead based method then tested for the presence of *M. ulcerans* using a TaqMan quantitative polymerase chain reaction (qPCR) targeting insertion sequence (IS) *2404* detected using a FAM labelled probe. The IS*2404* qPCR is multiplexed with an exogenous internal positive control (IPC) that uses a VIC probe, which assists in distinguishing a true negative reaction from one that has no result due to PCR inhibition.

**Weight (mg)**	**Replicate 1**		**Replicate 2**		**Replicate 3**	
	IS*2404* qPCR (Ct)	IPC (Ct)	IS*2404* qPCR (Ct)	IPC (Ct)	IS*2404* qPCR (Ct)	IPC (Ct)
10	20.464	26.337	19.681	26.484	18.747	26.262
20	19.393	26.339	17.473	26.194	17.974	26.269
40	16.912	25.994	16.409	26.371	16.389	26.354
60	16.497	26.234	16.066	26.495	15.697	26.332
80	15.406	27.224	15.437	27.93	15.06	26.551
90	15.283	26.084	14.741	24.805	14.808	26.396
100	14.383	26.016	14.315	26.772	15.218	26.142
110	Undetermined	Undetermined	Undetermined	Undetermined	Undetermined	Undetermined

Ct: cycle threshold.

Supplementary Table 2. Collection site and IS*2404* qPCR cycle threshold (Ct) values for possum excreta samples. Possum excreta surveillance samples were divided into two equal halves (by weight) with one half processed by the 'gold standard' PowerSoil Pro kit and the other half our SPRI-bead method. Both DNA extractions were then tested by IS*2404* qPCR to compare the efficiency between methods.

Sample number	Collection site	PowerSoil (Ct)	SPRI-bead (Ct)
1	RES19-02287	23.368	22.955
2	RES19-02289	32.526	36.738
3	RES19-02292	30.603	36.299
4	RES21-00902	24.711	24.387
5	RES21-00921	17.789	21.445
6	RES21-00930	36.297	36.008
7	RES21-00993	34.425	34.710
8	RES21-00999	19.563	22.043
9	RES21-01067-A	22.414	25.923
10	RES21-01067-B	27.638	32.006
11	RES21-01079	23.546	27.035
12	RES21-01131-A	28.779	31.029
13	RES21-01131-B	30.551	33.095
14	RES21-02262	ND	38.109
15	RES21-02291	31.141	34.312
16	RES21-02636	34.218	35.767
17	RES21-02644	37.100	34.285
18	RES21-02662	ND	34.144
19	RES21-03270	35.033	34.854
20	RES21-03330	35.620	33.125
21	RES21-03709-A	32.966	33.240
22	RES21-03709-B	33.671	35.769
23	RES21-03709-C	39.889	34.683
24	RES21-03709-D	28.173	28.850
25	RES21-03709-E	32.408	34.760
26	RES21-03709-F	33.385	34.117
27	RES21-03709-G	32.916	33.827
28	RES21-03709-H	32.714	33.757
29	RES21-03709-I	32.763	34.727
30	RES21-03709-J	38.367	35.132
31	RES21-03709-K	31.143	32.427
32	RES21-03709-L	33.119	39.067
33	RES21-03709-M	34.960	39.310
34	RES21-03709-N	32.405	39.748
35	RES21-03709-O	31.418	34.034
36	RES21-03709-P	32.749	34.810
37	RES21-03709-Q	32.763	33.667
38	RES21-03709-R	33.037	34.788
39	RES21-03709-S	33.073	35.226
40	RES21-03709-T	32.783	34.837
41	RES21-03709-U	33.755	34.927
42	RES21-03709-V	ND	34.012
43	RES21-03709-W	32.331	34.200
44	RES21-03709-X	32.099	33.210
45	RES21-03709-Y	32.600	33.717
46	RES21-03841	22.763	24.462
47	RES21-03885	28.341	23.455
48	RES21-03895	31.163	ND
49	RES22-03855-A	ND	ND
50	RES22-03855-B	ND	ND
51	RES22-03855-C	ND	ND
52	RES22-03855-D	ND	ND
53	RES22-03856-A	ND	ND
54	RES22-03856-B	ND	ND
55	RES22-03856-C	ND	ND
56	RES22-03856-D	ND	ND
57	RES22-03856-E	ND	ND
58	RES22-03856-E	ND	ND
59	RES22-03856-F	ND	ND
60	RES22-03856-G	ND	ND
61	RES22-03856-H	ND	ND
62	RES22-03856-I	ND	ND
63	RES22-03856-J	ND	ND
64	RES22-03856-K	ND	ND
65	RES22-03856-L	ND	ND
66	RES22-03856-M	ND	ND
67	RES22-03856-N	ND	ND
68	RES22-03856-O	ND	ND
69	RES22-03856-P	ND	ND
70	RES22-03856-Q	ND	ND
71	RES22-03856-R	ND	ND
72	RES22-03856-S	ND	ND
73	RES22-03856-T	ND	ND
74	RES22-03856-U	ND	ND
75	RES22-03856-V	ND	ND
76	RES22-03856-W	ND	ND
77	RES22-03856-X	ND	ND
78	RES22-03856-Y	ND	ND

ND: not detected.

Supplementary Table 3. Comparison of the efficiency of a SPRI-bead method with the DNeasy Blood & Tissue kit for extracting *M. ulcerans* DNA from swabs sampled from Australian possums as determined by IS*2404* qPCR.

Possum number (ID)	Swabbed site	Blood & Tissue (Ct)	SPRI-bead (Ct)
1 (W379-23)	Multiple ulcers	ND	ND
2 (W381-23)	Multiple ulcers	ND	ND
3 (W386-23)	Multiple ulcers	ND	ND
4 (W387-23)	Multiple ulcers	ND	ND
5 (W380-23)	Multiple ulcers	19.836	19.814
6 (6/22)	Oral cavity	36.346	34.806
	Cloaca	29.675	27.849
7 (13/22)	Oral cavity	39.652	36.017
	Cloaca	ND	ND
8 (14/22)	Oral cavity	31.072	25.043
	Cloaca	32.946	32.578
	Pouch	33.367	33.022
9 (15/22)	Oral cavity	34.952	ND
10 (20/23)	Oral cavity	33.762	32.982
	Cloaca	34.979	32.982
	Ulcer (L hind paw)	23.931	19.301

Ct: cycle threshold, ND: not detected, L: left.

REFERENCES

1. Doig KD, Holt KE, Fyfe JA, Lavender CJ, Eddyani M, Portaels F, Yeboah-Manu D, Pluschke G, Seemann T, Stinear TP. 2012. On the origin of *Mycobacterium ulcerans*, the causative agent of Buruli ulcer. BMC Genomics 13:258.
2. Williamson D, Howden B, Stinear T. 2017. *Mycobacterium chimaera* spread from heating and cooling units in heart surgery. N Engl J Med 376:600-602.
3. Stinear TP, Seemann T, Harrison PF, Jenkin GA, Davies JK, Johnson PD, Abdellah Z, Arrowsmith C, Chillingworth T, Churcher C, Clarke K, Cronin A, Davis P, Goodhead I, Holroyd N, Jagels K, Lord A, Moule S, Mungall K, Norbertczak H, Quail MA, Rabbinowitsch E, Walker D, White B, Whitehead S, Small PL, Brosch R, Ramakrishnan L, Fischbach MA, Parkhill J, Cole ST. 2008. Insights from the complete genome sequence of *Mycobacterium marinum* on the evolution of *Mycobacterium tuberculosis*. Genome Res 18:729-41.
4. Mohan A, Padiadpu J, Baloni P, Chandra N. 2015. Complete genome sequences of a *Mycobacterium smegmatis* laboratory strain (MC2 155) and isoniazid-resistant (4XR1/R2) mutant strains. Genome Announc 3(1):e01520-14.
5. Lee JYH, Carter GP, Pidot SJ, Guerillot R, Seemann T, Goncalves da Silva A, Foster TJ, Howden BP, Stinear TP, Monk IR. 2019. Mining the methylome reveals extensive diversity in *Staphylococcus epidermidis* restriction modification. mBio 10:e02451-19.
